# Global Renewable Energy Infrastructure Resilience Under Climate Risks

**DOI:** 10.1111/risa.70273

**Published:** 2026-06-23

**Authors:** Jingke Hong, Yang Chen, Wen Yi, Hung‐lin Chi

**Affiliations:** ^1^ School of Management Science and Real Estate Chongqing University Chongqing China; ^2^ Department of Building and Real Estate The Hong Kong Polytechnic University Hong Kong China

**Keywords:** climate risks, dynamic panel model, infrastructure resilience, renewable energy

## Abstract

Accelerating global climate risks increasingly threaten renewable energy infrastructure (REI). However, little evidence on heterogeneous impacts of climate risks on REI across countries, the moderating role of REI resilience, and post‐disaster recovery patterns is available, despite their critical importance for guiding resilient energy transitions and informing disaster risk governance. To address these issues, we employed dynamic panel models in 215 countries and regions from 2004 to 2022. We find that (1) climate risk significantly damages global REI, with disaster frequency and institutional resilience having mitigation effects. (2) The damage follows an inverted U‐shape with increasing disaster frequency and an “N” shape with increasing disaster duration. As renewable energy generation share increases, the damage intensifies and progresses through four increasingly severe stages. (3) Economic resilience exhibits a “Creative destruction” effect in developed nations and a “Build back better” recovery in poor countries. (4) Although social resilience worsens climate disaster damage globally, high disaster frequency and institutional resilience can facilitate a “Recovery to trend” in socially advanced nations. (5) REI in South America is the most affected, followed by Asia and Africa, whereas Europe is the least impacted. Wind energy is the most vulnerable, followed by bioenergy, solar, and hydropower.

## 1 Introduction

**FIGURE 1:**
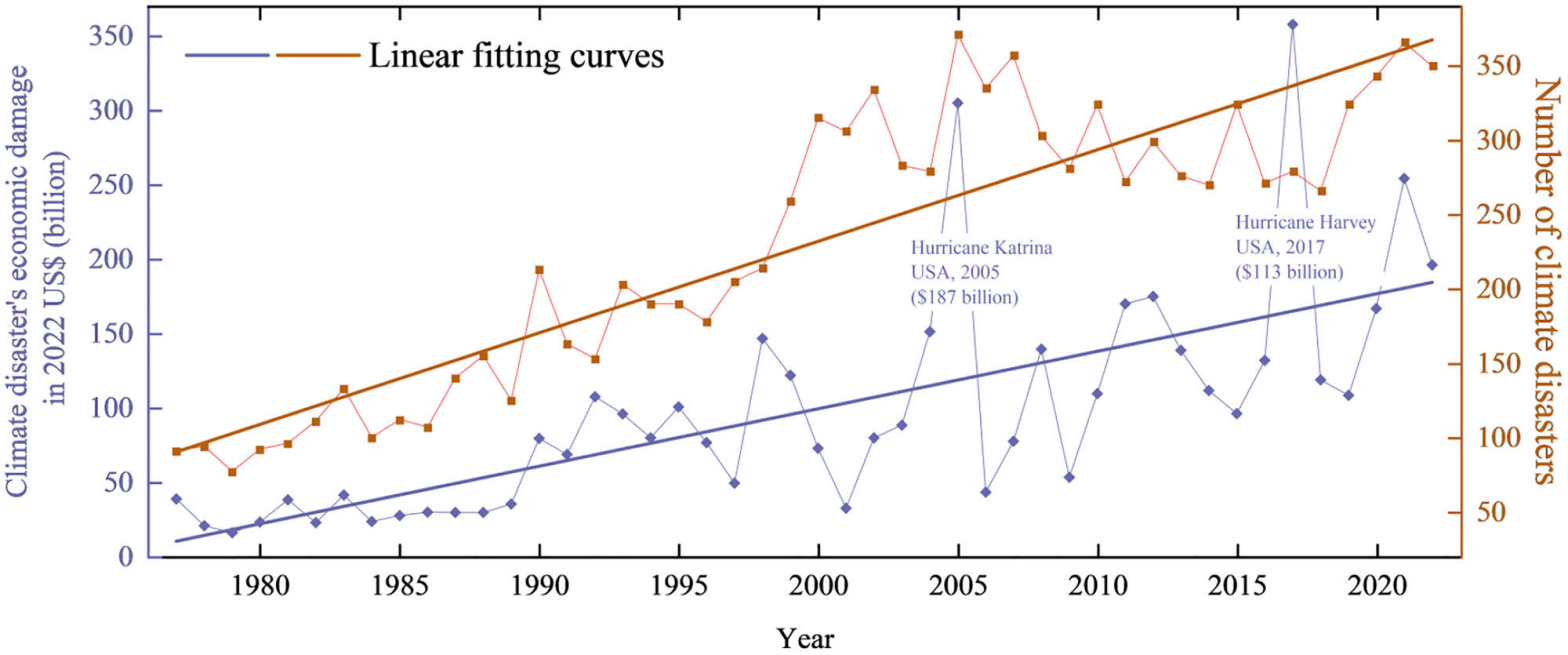
Increasing trends of global climate disasters in frequency and damage. (1) Climate disasters include drought, temperature extremes, storms, and floods and (2) data calculated by the authors using raw data from EM‐DAT, CRED.^2^

**FIGURE 2:**
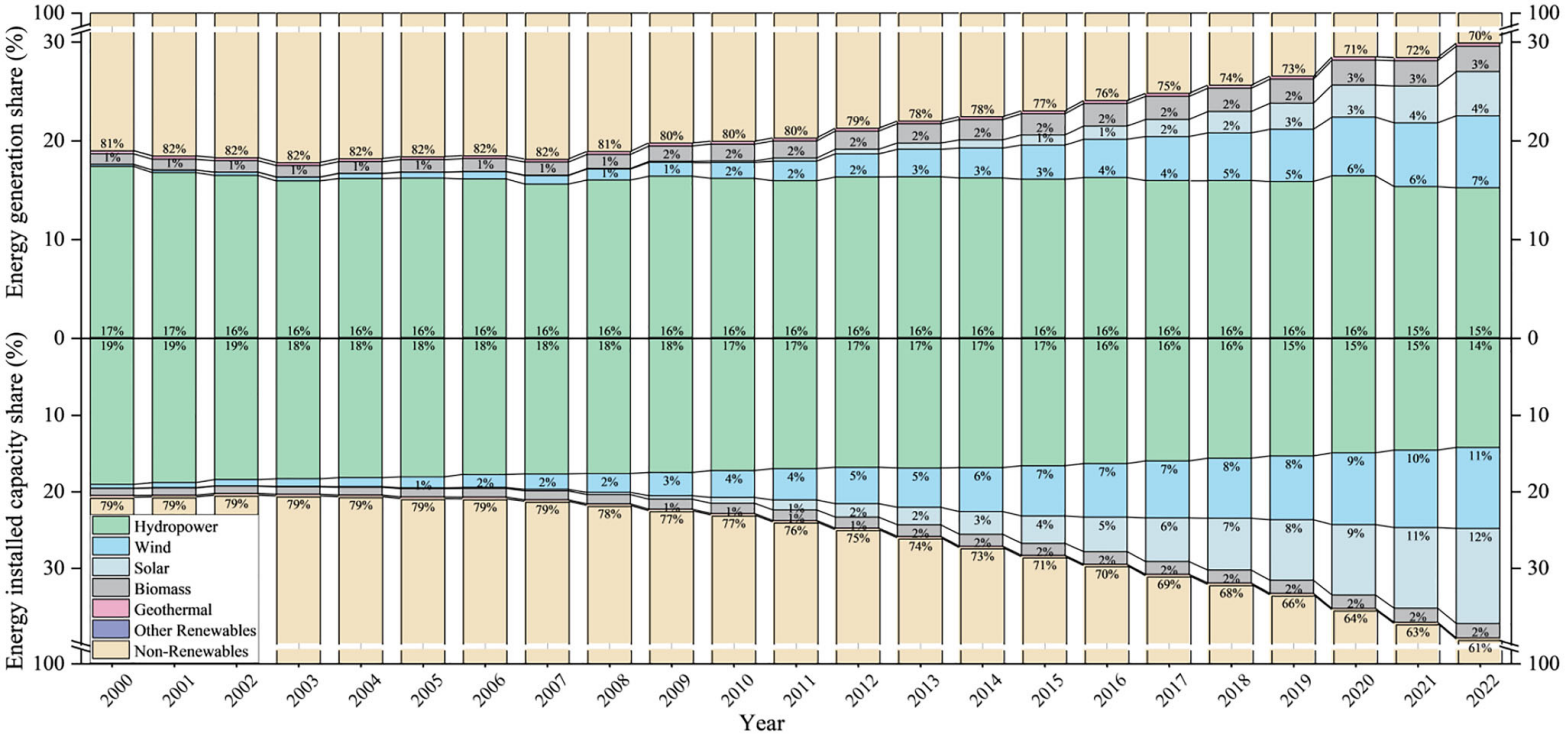
Development trends of RE generation and installed capacity. Data sourced from EIA.^3^

Accelerating climate change has intensified the frequency and severity of climate risks in recent decades (Song et al. 2023). Data from EM‐DAT (CRED 2024) (Figure 1) show a yearly increase in the number and economic losses of global climate disasters from 1977 to 2022, totaling over 10,000 extreme events and $4.47 trillion in economic losses. In response, nations globally have adopted renewable energy (RE) as a mitigation strategy. The share of RE in the energy system, including installed capacity and power generation, has gradually increased worldwide (Figure 2). The International Renewable Energy Agency (IRENA) projects that 90% of the world's electricity will come from renewable sources by 2050 (IRENA 2023). The concurrent trends of increasing climate risk and rising RE share underscore the critical need to enhance the climate resilience of renewable energy infrastructure (REI).

REI includes systems and facilities used to generate, store, and distribute energy from renewable sources. Infrastructure resilience refers to the capacity to withstand natural hazards (Marzi et al. 2019) and return to pre‐disaster equilibrium (Khan et al. 2022). However, RE systems, due to their dependence on natural resources and intermittent nature, are particularly vulnerable to extreme climate events (Chen et al. 2024). Hydropower, wind power, solar power, and biomass energy, which collectively account for over 98% of RE generation (EIA 2024), are sensitive to climate variables such as rainfall, wind speed, sunlight, and humidity (Wang et al. 2014). For example, droughts can severely limit hydropower generation, whereas floods can disrupt their normal operations (Lee et al. 2022). The reliance on natural resources makes RE generation intermittent and unstable, exposing it to climate risks (Park and Hur 2018) and prolonged power outages (Bagheri et al. 2019). Consequently, climate risk increases the uncertainty of RE and diminishes its performance and reliability (Fant et al. 2020). Additionally, heightened climate risk can raise financing costs for RE projects, potentially reducing (Chen et al. 2024) or interrupting (Agostino 2024) investments, challenging the deployment of RE technologies (Dell et al. 2014). Overall, climate risk can negatively impact energy‐related investments (Chen et al. 2021), innovation (Chen et al. 2024), efficiency (Okolo and Wen 2022), deployment (Oh and Oetzel 2011), and recovery (Zhao et al. 2024) through direct physical damage and indirect cascading effects (see Tables S1 and S2).

Although climate risk can damage REI, it can also raise awareness about disaster resilience among the public (Agostino 2024), businesses (Benincasa et al. 2024), and governments (Solecki et al. 2019). For instance, Solecki et al. (2019) found that hurricanes can enhance future resilience and increase government support for RE deployment. Li et al. (2023) found that natural hazards positively impacted Bangladesh's sustainable development in both the short and long run. Therefore, it is worth investigating how to enhance the disaster resilience of REI and leverage the experience of climate disasters to reduce future damage, adhering to the principle of “never let a good crisis go to waste” (Agostino 2024).

The literature on REI resilience can be broadly categorized into two strands: definition and assessment. There is no unified conclusion on the qualitative criteria of REI resilience. Although the IPCC (2011) proposed six elements of system resilience—economic resources, technology, information and skills, infrastructure, institutions, and equity (Smit and Pilifosova 2003)—subsequent studies have introduced other assessments, such as the “Adaptive Capacity Wheel” (Gupta et al. 2010), the four stages of preparation, absorption, recovery, and adaptation (Roege et al. 2014), internal and external resilience (Labaka et al. 2015), and even an index system including up to 196 evaluation indicators (Sharifi and Yamagata 2016). However, given the data unavailability, quantitative studies are often limited to micro‐systems (Bagheri et al. 2019) or regional levels (Wang et al. 2014).

Given the lack of a dedicated assessment framework for REI resilience in the current literature, this article synthesizes current research on system resilience (Borsekova et al. 2018; Gatto and Drago 2020; Labaka et al. 2015) and divides the unmeasured REI resilience into four dimensions: environmental, institutional, economic, and societal. We employ dynamic panel models to analyze the impact of global climate disaster events on REI across 215 countries and regions from 2004 to 2022 and examine the mitigating effects of REI resilience on disaster damage from these four dimensions. Additionally, we explore the heterogeneity of these dimensions across different countries and types of RE. The potential contributions of this article are as follows.

1. We address a research gap by empirically estimating the global impact of climate risk on REI. Despite the existing research has examined the negative effects of natural hazards on infrastructures and RE (Chen et al. 2024; Rahman 2018; Taghizadeh‐Hesary et al. 2021), the relevant studies still ignore the physical damages of climate disasters on REI which directly determines RE production and deployment. Moreover, previous studies have been limited in scope, often focusing on specific climate events (Hsiang and Jina 2014), projects (Bagheri et al. 2019), or regions (Wang et al. 2014), which may underestimate the full impact of climate risk by overlooking potential interactions and amplification effects between different types of extreme climate events (as shown in Table S2). Given that climate risk and RE goals are global issues requiring national‐level actions, this article employs dynamic panel data of 215 major economies using climate disaster data from EM‐DAT (CRED 2024) between 2004 and 2022. This approach allows for a comprehensive and global analysis of climate risk impact on REI across diverse economies.
2. This study also addresses the understudied moderating role of REI resilience in mitigating the impacts of climate risk on REI, as few studies have quantitatively estimated the relationship between REI resilience and natural hazards (Esteban and Portugal‐Pereira 2014) or climate risks (Xu et al. 2024). We synthesize current REI resilience research and analyze its mitigating effects and national heterogeneity across four dimensions: environmental, institutional, economic, and societal. This comprehensive analysis fills a gap in understanding REI resilience under climate disasters and provides policy‐relevant insights for countries developing REI.
3. Current research identifies four post‐disaster recovery patterns: “No recovery,” “Recovery to trend,” “Build back better,” and “Creative destruction” (Hsiang and Jina 2014). However, quantitative studies on these recovery types for national REI remain limited. We proposed quantitative criteria based on theory and analyzed REI recovery patterns across countries, offering a reference for post‐disaster REI recovery.

The article is structured as follows: Section 2 reviews the relevant literature. Section 3 outlines the methods and data. Section 4 reports the results. Section 5 discusses the findings. The final section concludes.

## 2 Literature Review

Resilience refers to a system's ability to absorb disturbances while maintaining its core functions, structure, identity, and feedback (Borsekova et al. 2018). However, the resilience of REI lacks a universally accepted definition. Contemporary resilience theory distinguishes engineering resilience (speed of return to equilibrium) (Jesse et al. 2024) from social‐ecological resilience (persistence across multiple stable states with learning and adaptation) (Folke 2006), emphasizing adaptive and transformative capacities governed by institutions and feedbacks. For energy infrastructure, resilience frameworks delineate system capacities into plan/prepare, absorb, recover, and adapt (Roege et al. 2014). Energy system resilience includes cascading interdependencies, cyber‐physical risks, and multistage recovery (Jasiūnas et al. 2021). For renewables specifically, variability and correlated weather risks pose system integration challenges (e.g., balancing variable nonfossil and conventional generation within the grid) and resource adequacy challenges (e.g., ensuring supply reliability despite renewable intermittency) at high penetration shares of variable RE (Heptonstall and Gross 2021). Conversely, modularity and distributed siting can reduce single‐point failures and aid operations (Nawaz Khan and Ali Abbas Kazmi 2022), whereas system‐level flexibility can lower integration costs and enhance restorative capacity after shocks (Hirth et al. 2015).

Complementing resilience theory, vulnerability models conceptualize risk as a function of exposure, sensitivity, and adaptive capacity (Wang et al. 2014). They quantify which components are more susceptible under given conditions, thereby providing operational indicators and assessment frameworks for defining and measuring resilience (Shen et al. 2024). Specifically, exposure primarily captures the environmental dimension, which is the physical proximity of climate disaster and energy system (Jasiūnas et al. 2021) and thus defines the magnitude of potential shock. Sensitivity describes how strongly system components and social groups are affected by a hazard (Niklas and Mey 2023). As this depends on socio‐economic conditions, sensitivity spans the social and economic dimensions of resilience. Adaptive capacity is the ability to anticipate, absorb, reorganize, and recover (Beyza and Yusta 2021) and can be further categorized into three distinct domains: institutional, economic, and societal capacities. For example, institutional capacity, comprising governance, planning, and regulation, facilitates coordinated prevention and response Gatto and Drago (2020). Economic capacity, encompassing fiscal space, insurance, market mechanisms, and investment, determines repair and reconstruction potential (Gasser et al. 2021). Societal capacity, including social capital and local organization, shapes preparedness, informal coping, and recovery dynamics (Niklas and Mey 2023). Therefore, conceptualizing environmental, institutional, economic, and societal resilience as distinct yet interacting dimensions maintains the underlying exposure–sensitivity–adaptive capacity logic, while also providing a theoretically grounded bridge from abstract constructs to the empirical indicators employed in this study.

### 2.1 Environment Resilience

Disaster damages can be influenced by environmental resilience characteristics (Ward and Shively 2017), which can be divided into external and internal environments. Exposure to climate hazards, including their frequency and duration, builds disaster experience, facilitating REI resilience in response to external pressures. The development and diversity of a country's RE system represent the disaster resilience of its internal environment.

#### 2.1.1 External Environment

The public (Myers et al. 2013) and policymakers (Porter et al. 2015) generally prefer forming inclinations based on directly observed trends and events rather than abstract climate change information. Because experience‐based disaster information, especially seasonal flood disasters (Spence et al. 2011), is more readily understood (Ogunbode et al. 2017) and triggers action (Marx et al. 2007). Besides, climate forecasts are inherently uncertain and limited in detail (Porter et al. 2015).

The impact of disaster experience on climate resilience remains inconclusive. Some studies suggest disasters significantly shape resilience (Reckien et al. 2018), whereas others find no relationship (Yeganeh et al. 2020). Experiences of extreme weather events can enhance public willingness to pay for climate mitigation (Gould et al. 2024) and mitigation actions (Shao and Hao 2024). However, frequent major disasters often have a limited impact on urban adaptation actions. As the frequency of a particular disaster increases, government preparedness for other disasters may decrease (Nohrstedt et al. 2022). In other words, if multiple disasters occur simultaneously, experience from a specific disaster may not suffice for new types of disasters. Evidence shows that individuals with prolonged disaster experience perceive greater risks (Dai et al. 2015) and take preemptive energy measures (Amin et al. 2021), but more experience does not always improve personal disaster preparedness (Baik et al. 2020), whereas governments with a history of natural hazards respond quickly in the short term but also require flexible disaster management plans for long‐term effectiveness (Li et al. 2023). Overall, these conflicting views largely stem from studies limited to specific countries or regions, lacking global evidence and consideration of national heterogeneity (Nohrstedt et al. 2022).

#### 2.1.2 Internal Environment

The intermittency of RE generation can make electrical systems vulnerable to climate disasters (Beyza and Yusta 2021), potentially causing prolonged power outages (Baik et al. 2020). However, this vulnerability can be mitigated through energy portfolio diversification (Kosai and Unesaki 2020) and storage systems (Esteban and Portugal‐Pereira 2014).

Energy diversity is crucial for long‐term energy security (Esteban and Portugal‐Pereira 2014). Over‐reliance on a single resource increases the risk of energy supply disruptions (Chuang and Ma 2013). Despite this, diversified energy sources can ensure that highly dependent energy systems are not at high risk (Bhattacharyya 2009) and can effectively respond to external pressures like market fluctuations and supply shortages (Chuang and Ma 2013). Although traditional backup generators (diesel and natural gas) may not mitigate long‐term climate interruptions (Bagheri et al. 2019), sustainable solutions (Gonzalez et al. 2023) like hybrid RE systems (Thirunavukkarasu et al. 2023) and battery storage systems (Esteban and Portugal‐Pereira 2014) can enhance resilience to prolonged outages. For example, battery storage systems can help Japan's 100% RE system achieve post‐disaster recovery (Esteban and Portugal‐Pereira 2014).

### 2.2 Institutional Resilience

Institutions, including governance and management structures, are key determinants of climate change adaptation (Taghizadeh‐Hesary et al. 2021) and critical infrastructure resilience (Huddleston et al. 2023). The impact of any disaster depends on the institution's ability to respond and recover (Borsekova et al. 2018). High‐quality institutions can reduce resilience costs, enhance disaster prevention and response (Khan et al. 2022), and minimize economic losses and recovery time (Agostino 2024), even in underdeveloped countries (Spence 2004). Stable and democratic political institutions (Sequeira and Santos 2018) ensure the effective implementation of RE support policies (Chen et al. 2024), potentially mitigating negative climate change impacts (Shahbaz et al. 2022). Conversely, institutional corruption (Cadoret and Padovano 2016) and lack of political support (Porter et al. 2015) can hinder RE deployment. However, the importance of policy institutions for climate adaptation actions remains controversial (Nohrstedt et al. 2022), with many studies attributing this to national heterogeneity (Cinner et al. 2018; Williams et al. 2015).

### 2.3 Economic Resilience

Economic resilience denotes the inherent and adaptive responses that allow households, firms, markets, and regions to avert potential losses during and after disasters (Rose 2004). It is the fraction of losses avoided relative to a maximum potential loss (Rose 2007). By mobilizing behavioral adjustments, market re‐equilibration, and financial responses, resilience dampens the translation of physical shocks into economic damage. Therefore, models that omit it overstate losses and misvalue recovery capacity (Rose 2017). Economic development expands the resource buffers and adjustment margins that underpin these mechanisms and is thus central to recovery (Chen et al. 2024). Empirically, higher income (Albuquerque and Rajhi 2019) and capital (Mavhura et al. 2021) are associated with greater disaster resilience (Khan et al. 2022). A stable economic environment helps countries recover quickly from climate disasters (Chen et al. 2024) and mitigate negative impacts on RE (Z. Wang, Zhang, et al. 2023). Conversely, economic crises and high policy costs can lead to the abolition of RE measures (Prontera 2021).

Countries with strong financial foundations and high incomes suffer less from disasters than credit‐constrained countries (McDermott et al. 2014), prepare more adequately for disasters (Noy 2009), and reduce vulnerability to climate change (Li et al. 2023). Low‐income countries, lacking environmental reforms and climate adaptation policies, are disproportionately affected by natural hazards (Noy 2009). Disaster losses can impact their infrastructure and RE by reducing foreign exchange reserves (Khan and Anwar 2022). Poorer regions exhibit higher vulnerability to climate change impacts on RE due to higher opportunity costs of disaster prevention policies (Wang et al. 2014). As countries develop economically, they allocate more resources to reduce disaster risks (Toya and Skidmore 2007) but may compromise on infrastructure quality due to cost constraints (Rahman 2018).

There are no consistent results on the role of the economic environment in disasters, primarily due to national heterogeneity (Hsiang and Jina 2014). Therefore, the role of economic heterogeneity in the impact of climate risk on REI requires investigation.

### 2.4 Social Resilience

Social resilience to climate disasters refers to communities’ capacity to withstand, adapt to, and recover from climate events (Saja et al. 2018). Regions with high social resilience demonstrate greater cohesion and inclusiveness, facilitating coordinated economic and social policies (Borsekova et al. 2018). While wealthier societies generally possess superior disaster recovery capabilities, they may also face greater disaster risks due to higher exposure (Nohrstedt et al. 2022). Empirical analysis suggests that social development has a limited short‐term impact on RE consumption (Khribich et al. 2021). The varying capacities to manage disaster risks across regions are attributed to significant heterogeneity in social development levels (Song et al. 2024), highlighting the complex relationship between social factors and climate disasters.

### 2.5 Interactive Dynamics Among Resilience Dimensions

The realization of REI outcomes depends on how environmental, institutional, and societal factors interact with economic capacity. On the environmental side, adequate financial resources enable portfolio diversification and flexibility, which reduces exposure to correlated hazards and aids restoration (Chuang and Ma 2013; Huddleston et al. 2022). On the institutional side, effective policies and competent regulators mobilize private capital for flexibility and storage upgrades, whereas institutional weaknesses amplify policy risk and stall investment (Cadoret and Padovano 2016). On the societal side, household resources and community cohesion condition preparedness and the ability to cope with prolonged outages, influencing both recovery costs and the distribution of welfare losses, while enhancing the resilience of socially vulnerable groups can reduce these economic burdens (Carvallo et al. 2022; Dugan et al. 2023; Siegel et al. 2024).

Additionally, social resilience interacts with environmental and institutional factors in determining outcomes for REI. For example, where hazard exposure intersects with social vulnerability, outage durations lengthen and recovery trajectories diverge across communities, which in turn influences public support for resilience investment (Ganz et al. 2023; Gonzalez et al. 2023). Nohrstedt et al. (2022) contend that the recurrence of disasters alone is insufficient for adaptation and that effective adaptation relies on institutional governance to transform experience into learning and policy change. Finally, the distribution of social resources influences the adoption of protective measures, and energy system investments are most effective when paired with community‐level strategies that improve access and participation (Carvallo et al. 2022; Siegel et al. 2024).

## 3 Methods

### 3.1 Model

#### 3.1.1 Impact of Climate Risk on REI

We employ a dynamic panel model to examine the impact of climate risk on REI. A one‐period lag of REI is used as an independent variable to capture the historical accumulation effects, reflecting how current levels are influenced by past investments. This approach also addresses endogeneity concerns arising from omitted variables and potential reverse causality. To mitigate bias, we control for relevant variables, as well as time‐fixed effects (varying over time but constant across countries), country‐fixed effects (varying across countries but constant over time), and country‐specific time trends. We apply clustered robust standard errors to account for heteroscedasticity. The baseline model is

(1) $$\begin{array}{ccc} \mathrm{lnRE}I_{it} & = & \varphi_{1}L.\mathrm{lnRE}I_{it}+\mu_{1}\mathrm{lnCD}{R_{i}}_{t}+\lambda \mathrm{control}s_{it}\\ & & +\;\delta_{t}+\eta_{i}+\mathrm{id}\times \mathrm{tren}d_{it}+\alpha_{0}+\varepsilon_{it} \end{array}$$

where REI and CDR represent the levels of renewable energy infrastructure and climate risks, respectively. Controls represent the vector of control variables, $\delta_{t}$, $\eta_{i}$, and $\mathrm{id}\times \mathrm{tren}d_{it}$ represent time‐fixed effects, country‐fixed effects, and country‐specific time trends, respectively, whereas $\alpha_{0}$ and $\varepsilon_{it}$ represent the constant and the error term. The analysis spans 2004–2022, a period marked by heightened global attention to RE following the 2004 Bonn Conference.

#### 3.1.2 Moderating Effect of REI Resilience

To assess how REI resilience moderates the relationship between CDR and REI, we extend the baseline model as follows:

(2) $$\begin{array}{ccc} \mathrm{lnRE}I_{it} & = & \varphi_{1}L.\mathrm{lnRE}I_{it}+\mu_{k}\mathrm{lnCD}{R_{i}}_{t}+\beta_{k}\mathrm{REI}{R_{ki}}_{t}+\varphi_{k}\ln\mathrm{CD}{R_{i}}_{t}\\ & & \times \;\mathrm{REI}{R_{ki}}_{t}\;+\lambda \mathrm{control}s_{it}+\delta_{t}+\eta_{i}+\mathrm{id}\times \mathrm{tren}d_{it}\\ & & +\;\alpha_{0}+\varepsilon_{it} \end{array}$$

where REI resilience represents four resilience dimensions of environmental, institutional, economic, and social, and $\varphi_{k}$ represents the moderating effect.

#### 3.1.3 REI Recovery Patterns

Following Hsiang and Jina (2014), we identify four possible post‐disaster trajectories for REI: (1) “No recovery”: Infrastructure remains persistently below its pre‐disaster path, as fiscal stress and higher risk premia depress investment for years, leaving REI development lagging behind; (2) “Recovery to trend”: Temporary losses are erased as investment resumes and REI eventually rejoins the pre‐disaster growth path; (3) “Build back better”: Reconstruction improves quality and reduces future risks. The Sendai Framework defines it as using recovery and reconstruction to raise standards and reduce future risk without necessarily changing the economic structure (UNISDR 2015); (4) “Creative destruction”: Obsolete capital is replaced by higher productivity technologies through structural reallocation, delivering the highest post‐disaster REI levels and exceeding the “Build back better” outcome. This trajectory emerges when disasters accelerate capital turnover, embed superior vintages of renewables, storage, and controls, exploit learning curves and deployment‐driven cost declines (Way et al. 2022), and enable system‐wide network reoptimization rather than mere hardening (Hallegatte and Dumas 2009). Hsiang and Jina (2014) emphasize that “Creative destruction” is conditional and requires combinations of economic and policy resilience to channel reconstruction into transformational upgrades.

**FIGURE 3:**
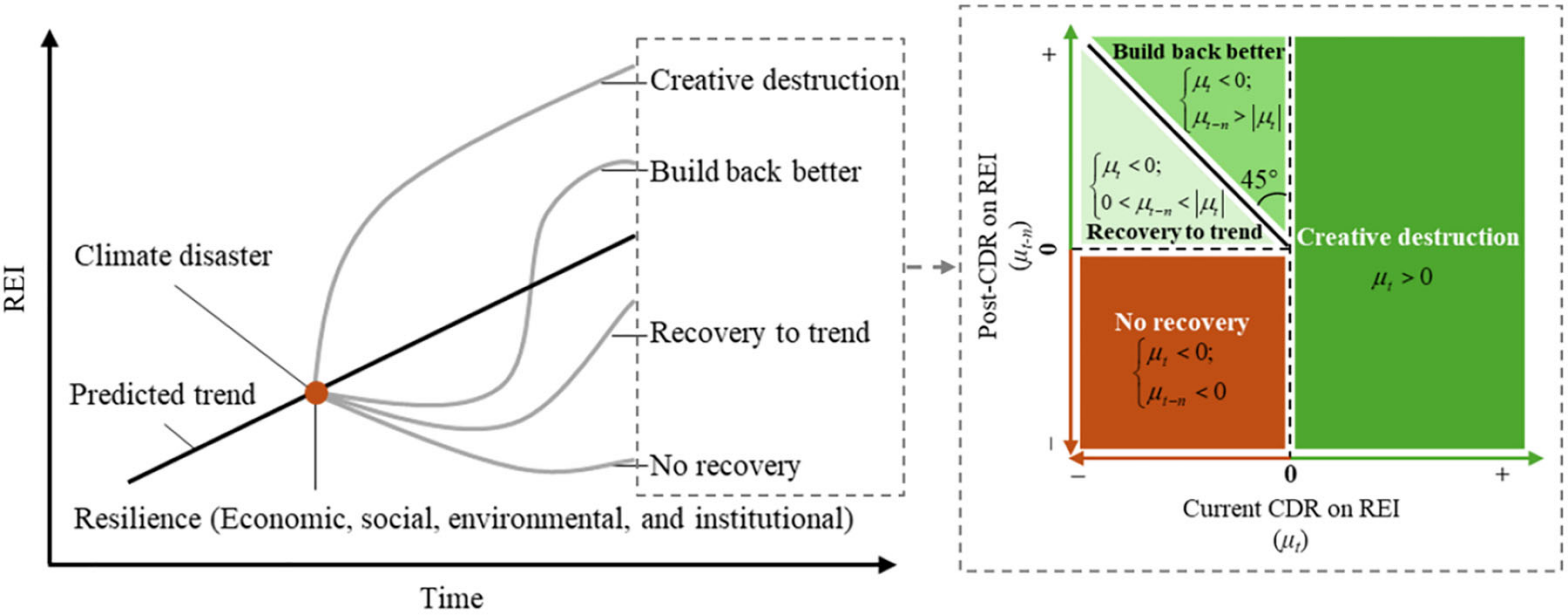
Recovery of REI after climate disasters. A negative value means significant damage to REI, whereas a positive value means recovery or promotion. REI, renewable energy infrastructure.

As shown in Figure 3, we use the current and lagged impacts of climate disasters on REI as the horizontal and vertical axes, respectively, to depict the four recovery trajectories. When the current impact (*μ_t_*) is positive, the REI trajectory lies above the predicted trend, corresponding to the “Creative destruction” pattern. When *μ_t_* is negative but the lagged impact (*μ_t__−__n_*) is positive and greater than |*μ_t_* |, post‐disaster recovery exceeds the initial damage, reflecting a “Build back better” outcome. Conversely, if *μ_t_* is negative and *μ_t__−__n_* is positive but smaller than |*μ_t_* |, the process represents a “Recovery to trend.” Finally, when both *μ_t_* and *μ_t__−__n_* are negative, the REI exhibits “No recovery.”

### 3.2 Variable Selection

#### 3.2.1 Dependent and Core Independent Variables

For REI, indicators adopted by previous research include installed capacity (Kersey et al. 2021), RE generation share (Ly 2025), and per capita RE generation (Calcaterra et al. 2024). Installed capacity reflects the scale of facilities but not generation efficiency or utilization. High capacity may not translate to high output due to technical or climatic conditions (Frew et al. 2021). The RE generation share also omits population effects, and a high share does not necessarily imply high generation capacity (Pappis et al. 2021). Per capita RE generation, by contrast, accounts for both total capacity and population, providing a comprehensive measure (Calcaterra et al. 2024). Thus, we use it as the proxy for REI. As a robustness check, we also use total RE generation as a proxy for REI. Geothermal power, minimally affected by climate risk and contributing less than 2% to total RE, is excluded. Our measure, therefore, aggregates solar, wind, hydro, and biomass, which together comprise over 98% of global RE output. Data come from IRENA's *Renewable Energy Statistics* (IRENA 2023), a dataset widely employed in cross‐country analyses (Egli et al. 2023).

The impact of climate risk is typically measured by economic losses, the proportion of the affected population, and the mortality rate (Lee et al. 2021; Rahman 2018). However, economic losses depend on the region's development level, distorting cross‐regional comparisons. UNDRR (2019)’s *Global Assessment Report* explains that increases in recorded monetary losses can stem from high values of exposed assets and should not be confused with high risk. The proportion of the affected population reflects disaster coverage but can overestimate severity when mildly affected individuals dominate. UNDRR (2017) states that “affected” includes people injured, ill, evacuated, or displaced, and those with disrupted livelihoods, which is a broad set that can swell counts even when most cases are minor. In contrast, the mortality rate directly captures the threat to life and provides a more objective measure of disaster severity (Felbermayr and Gröschl 2014). Therefore, we use the mortality rate to assess climate disaster severity. Climate disasters include floods (from heavy rain, glacier melt, hurricanes, etc.), storms, droughts, and extreme temperatures. Mortality rate is constructed as disaster deaths per country–year divided by total population. Deaths come from EM‐DAT (CRED 2024), a widely used database with standardized inclusion criteria (≥10 deaths, ≥100 affected/injured/homeless, or a state of emergency/international appeal), ensuring reliability (Delforge et al. 2025).

#### 3.2.2 Control Variables

To minimize confounding effects, we control for the following seven variables:

1. **Economic level (PGDP)**: Economic development provides the fiscal basis for infrastructure and deployment, and higher income and activity levels are strongly associated with RE expansion (Sadorsky 2009). Accordingly, per‐capita GDP is widely used as a key driver in empirical studies (Bourcet 2020). Wealthier nations possess more resources for REI, whereas poorer countries, facing resource constraints, prioritize disaster resilience over RE goals. We measure this using per capita GDP (constant 2015 US$). Data come from World Bank WDI (World Bank 2024), an internationally harmonized database with transparent sources and methods.
2. **RE endowment (L.REI)**: RE development is determined by a country's energy endowment. REI exhibits significant persistence and path dependence, as existing capacity and industrial chains drive subsequent additions. Therefore, including a one‐period lag in the dynamic panel (Berk et al. 2020) provides a reasonable way to capture such endogenous inertia (Bölük and Kaplan 2022).
3. **Energy consumption (EC)**: Per‐capita EC reflects underlying demand and pressures for electricity expansion, thereby spurring substitution and incremental REI (Damette and Marques 2019). Panel evidence for Asian countries also shows a statistically significant link between EC and renewable use (Hoa et al. 2023). We measure this using per‐capita EC from the US Energy Information Administration (EIA 2024), the Department of Energy's independent statistical agency, whose indicators are widely used in policy and research.
4. **Population size (Pop)**: Population size directly raises electricity demand, creating strong energy expansion pressures (Zaman et al. 2012). A large population increases energy demand and supports economies of scale, promoting REI development. The data come from the World Bank's WDI (World Bank 2024).
5. **Openness (Open)**: Trade openness facilitates access to RE technology and capital, reducing the cost of REI development and promoting its expansion (Zhang et al. 2021). We use the trade share of GDP to measure openness, which comes from the World Bank's WDI (World Bank 2024).
6. **Foreign direct investment (FDI)**: FDI provides financial capital and introduces advanced technology and management practices critical for large‐scale RE projects (Tan et al. 2022). The data come from the World Bank's WDI (World Bank 2024).
7. **Industrial structure (IS)**: Energy‐intensive industries demand more energy, spurring REI development (Rissman et al. 2020). At the macro level, industrial infrastructure investment has been identified as an important positive driver of RE development (Y. Wang, Wang, et al. 2023). We use the proportion of industrial output to GDP as a proxy for IS. The data come from the World Bank's WDI (World Bank 2024).

#### 3.2.3 Moderating Variables

As discussed, we categorize REI resilience into environmental, institutional, economic, and social dimensions.

##### 3.2.3.1 Environmental Resilience

This dimension is further divided into external and internal resilience. External resilience is measured by the frequency and average duration of climate disasters, as recurrent and prolonged events enhance disaster experience (Onuma et al. 2017), thereby strengthening subsequent adaptation (Van Loon et al. 2024). Frequent exposure to disasters compels governments, businesses, and individuals to refine risk management strategies, thereby improving their preparedness for future events. Consequently, higher frequencies and longer durations may strengthen external environmental resilience. The data come from EM‐DAT (CRED 2024). Internal resilience is represented by the RE power generation share in total energy. A high RE share indicates advanced RE processes but also highlights potential energy security risks due to over‐reliance on renewables (Esteban and Portugal‐Pereira 2014). The data come from IRENA (2023).

##### 3.2.3.2 Institutional Resilience

This is captured by the incentives and regulatory support index for RE. Unlike broad institutional indicators such as corruption control and government efficiency, this index directly reflects the support policies and institutions provide for RE development. Clear and stable policy instruments, such as feed‐in tariffs, renewable portfolio standards, and auctions, reduce risks and increase returns, thereby mobilizing private capital and accelerating REI deployment (Polzin et al. 2019). Institutional strength and policy stability are thus preconditions for renewable electricity growth and diffusion (Lyon 2016). We measure this factor using Regulatory Indicators for Sustainable Energy (RISE) (World Bank Group 2024), a World Bank policy scorecard with transparent, question‐based methodology and equal‐weight aggregation, and the renewables pillar averages indicator scores (0–100). It is updated in periodic global reports and used in peer‐reviewed research (Drago and Gatto 2022; Gatto and Drago 2020).

##### 3.2.3.3 Economic Resilience

Per‐capita GDP is a standard proxy for economic resilience. Richer countries have greater fiscal space, infrastructure quality, and administrative capacity to finance REI, preparedness, and recovery (Kahn 2005). Cross‐country evidence shows high income reduces disaster mortality and losses (Toya and Skidmore 2007), and that poorer economies suffer larger post‐disaster output declines (Noy 2009). IPCC (2011) also identifies economic wealth as a core determinant of adaptive capacity. Thus, per capita GDP serves as a proxy for a country's economic resilience to climate risk.

##### 3.2.3.4 Social Resilience

The Human Development Index (HDI) aggregates three social dimensions of health, education, and standard of living via a geometric mean, explicitly framing human development in social terms (UNDP 2024). These dimensions are the quintessential social dimensions, which underpin adaptive capacity. Therefore, HDI is a defensible proxy for social resilience in cross‐country analyses. Kaushik et al. (2024) suggest that high national HDI is associated with low disaster mortality, which reflects how improvements in HDI enhance a society's overall capacity to adapt and recover from disasters.

To maximize coverage across 215 countries and maintain a balanced panel, the data are from 2004 to 2022. For the missing 2022 RE share, we apply the average of the preceding 3 years, which is recommended by UN agencies for country indicators to reduce year‐to‐year noise (Food and Agriculture Organization 2019). Descriptive statistics for all variables considered in this study are presented in Table 1.

**TABLE 1:** Descriptive statistics.

Variable	Indicator	Measurement	Symbol	Obs	Mean	Std. Dev.	Min	Max
Dependent variable	RE infrastructure development	RE generation per capita	lnREI	3724	10.300	5.467	−4.605	17.546
Core variable	Climate risks	Mortality rate in disasters	lnCDR	2085	−4.560	0.185	−4.605	−1.224
Control variable	Economic level	GDP per capita	lnPGDP	3645	8.618	1.438	5.569	11.766
	RE endowment	Lagged lnREI	L.lnREI	3528	10.989	4.525	−4.605	17.546
	Energy consumption	Energy consumption per capita	lnEC	3503	6.569	1.950	−4.605	10.181
	Demographics	Population size	lnPop	3800	15.430	2.270	9.189	21.072
	Openness	Trade share of GDP	lnOpen	3218	4.351	0.515	0.997	6.761
	Foreign investment	Foreign direct investment	lnFDI	3500	1.012	1.363	−5.978	7.444
	Industrial structure	Industrial output share of GDP	lnIS	3511	3.141	0.490	1.018	4.462
Moderator	External environment resilience	Disaster frequency	Freq	2125	2.771	3.524	1	37
		Disaster duration	Dura	2125	30.089	88.873	0.250	1826
	Internal environment resilience	RE generation share	REGS	3838	32.505	32.866	0	100
	Institutional resilience	RE support index	lnIR	1584	3.227	1.338	−4.605	4.543
	Economic resilience	GDP per capita	lnPGDP	3645	8.618	1.438	5.569	11.766
	Social resilience	Human Development Index	HDI	3469	0.696	0.158	0.286	0.967

Abbreviations: RE, renewable energy; REI, renewable energy infrastructure.

## 4 Results

### 4.1 The Impacts of Climate Risk on REI

Table 2 presents the results from our empirical models. Regardless of whether control variables are included or whether three types of fixed effects (country, year, and country's time trend) are applied, all models (1–4) consistently demonstrate that global climate risk significantly damages REI at the 1% level. Specifically, a 1% increase in climate risk death rates is associated with a reduction in REI ranging from 1.93% to 9.78%.

**TABLE 2:** The impacts of climate risk on renewable energy (RE) infrastructure development.

Model	1	2	3	4
lnCDR	−5.4669^***^	−9.7758^***^	−1.9288^***^	−3.1112^***^
	(0.582)	(0.644)	(0.599)	(0.699)
L.lnREI		0.8600^***^		0.7988^***^
		(0.034)		(0.071)
lnPGDP		−0.4602^***^		0.4037
		(0.168)		(0.700)
lnEC		0.5913^***^		0.0460
		(0.155)		(0.460)
lnIS		−0.0649		−0.1345
		(0.296)		(0.376)
lnOpen		−0.8680^***^		−0.2657
		(0.244)		(0.342)
lnFDI		−0.0292		−0.0307
		(0.078)		(0.066)
lnPop		−0.2033^***^		2.3279^**^
		(0.073)		(1.128)
_cons	−14.0725^***^	−35.5615^***^	2.0718	−53.2356^***^
	(2.658)	(3.512)	(2.726)	(19.718)
Controls	No	Yes	No	Yes
Country effect	No	No	Yes	Yes
Year effect	No	No	Yes	Yes
Country's time trend	No	No	Yes	Yes
*R*_squared	0.0407	0.4193	0.8130	0.8810
*N*	2080	1619	2070	1610

*Note*: Clustering robust standard errors are in parentheses; ***, **, and * denote significance at 1%, 5%, and 10% levels; subsequent tables follow the same conventions.

Notably, when moving from Models 1 and 2 to Models 3 and 4 by incorporating three‐dimensional fixed effects, the *R* ^2^ increases, whereas several control variables that were significant in Model 2 lose statistical significance. This suggests that these fixed effects capture much of the variation previously explained by the control variables and other unobserved confounding factors. Consequently, we adopt Model 4 as the baseline for subsequent analyses.

### 4.2 Robustness Analyses

To ensure robustness, we controlled as many covariates as possible, employed three‐dimensional fixed effects, and utilized cluster‐robust standard errors. Furthermore, we conducted four robustness checks, as detailed in Table 3.

**TABLE 3:** Robustness analyses.

Method	SGMM	Replace *Y*	Replace *X*				Winsorize	Lag effects	
		REI→REG	CDR→Death	CDR→Loss1	CDR→Loss2	CDR→ZDR	(1%, 99%)	L.lnCDR	L.Controls
Model	1	2	3	4	5	6	7	8	9
lnCDR	−4.5466***	−5.4260***					−4.4626***	−3.9094***	−4.0806***
	(0.992)	(1.242)					(0.829)	(0.862)	(0.892)
lnDeath			−0.0629***						
			(0.018)						
Loss1				−0.0033					
				(0.016)					
Loss2					−0.0476				
					(0.039)				
ZDR						−0.1087*			
						(0.063)			
L.lnREI	0.8694***	0.2281***	0.7952***	0.7926***	0.7857***	0.7855***	0.8019***	0.8414***	0.6194**
	(0.040)	(0.074)	(0.071)	(0.070)	(0.077)	(0.077)	(0.071)	(0.088)	(0.267)
L.lnCDR								0.1023	0.3115
								(0.246)	(0.368)
Controls	Yes	Yes	Yes	Yes	Yes	Yes	Yes	Yes	L.Controls
Country effect	Yes	Yes	Yes	Yes	Yes	Yes	Yes	Yes	Yes
Year effect	Yes	Yes	Yes	Yes	Yes	Yes	Yes	Yes	Yes
Country's time trend	Yes	Yes	Yes	Yes	Yes	Yes	Yes	Yes	Yes
*R*_squared	/	0.8343	0.8765	0.8752	0.8603	0.8606	0.8828	0.8731	0.8734
*N*	1619	1610	1610	1610	1398	1398	1610	1174	1108

*Note*: (1) Robust standard errors are in parentheses; (2) for Model 1, the collapse option is set in SGMM; the *p* value of AR (2) is 0.268; the *p* value of the Hansen test is 0.217. (3) REG, Loss1, and Loss2 denote the total RE generation, per‐capita economic loss, and the economic loss per affected person, respectively. ZDR denotes the composite climate risk, constructed as the sum of the z‐standardized values of CDR, Loss1, and Loss2.

Abbreviations: REI, renewable energy infrastructure; SGMM, system generalized method of moments.

Although climate disasters are exogenous events posing minimal endogeneity concerns, using the mortality rate as a proxy for climate risk may still introduce endogeneity issues. For example, economic development could simultaneously affect mortality rates and REI during disasters. From a theoretical perspective, climate disasters typically precede their impact on REI, whether through direct physical damage or indirect channels, suggesting that reverse causality is unlikely. However, recent studies indicate that human activities can exacerbate climate change, increasing the frequency and intensity of climate risk, which raises concerns about potential reverse causality in our model.

To address this, we employed instrumental variable (IV) and used the dynamic panel system generalized method of moments (SGMM) estimation. Specifically, we used lagged REI and lagged climate risk as IVs. The deeper lags of REI are uncorrelated with the current error term while remaining highly correlated with the current REI. Similarly, past mortality rates are related to current mortality but are unlikely to directly influence current REI. To mitigate overfitting, we utilized the “collapse” option in Stata's “xtabond2” command to limit the number of IVs. The Arellano–Bond test results for Model 1 in Table 3 indicate that we cannot reject the null hypothesis of “no second‐order serial correlation,” providing evidence in support of our model specification. Additionally, the Hansen test for overidentifying restrictions suggests that we cannot reject the null hypothesis that “all instruments are valid,” further validating our instrument set. Together, these tests support the adequacy of our IVs. The result (−4.5466^***^) aligns with our baseline model findings.

Moreover, we performed additional robustness checks by (1) replacing the dependent variable (substituting per capita RE generation for total RE generation) (Model 2), (2) replacing the core variable by substituting the mortality rate (CDR) with the number of deaths (Death), per‐capita economic loss (Loss1), economic loss per affected person (Loss2), and a composite climate risk index (ZDR) combining CDR, Loss1, and Loss2 (Models 3–6); and (3) winsorizing the top and bottom 1% of extreme values (Model 7). Finally, we conducted a 1‐year lag analysis of CDR (Model 8) and a lagged analysis of the control variables (Model 9).^1^

We found that when disaster intensity is measured by per capita losses or losses per affected person, the coefficients are negative, as in the case of mortality rates, but statistically insignificant (Models 4 and 5). Neumayer and Barthel (2011) argue that global disaster‐related economic losses mainly reflect variations in wealth and defensive investments rather than disaster intensity itself, and thus, using such measures may lead to biased or insignificant estimates. Similarly, Felbermayr and Gröschl (2014) highlight that loss‐based indicators are inherently correlated with GDP, which may introduce estimation bias. This may explain why our results based on economic loss indicators yield negative but statistically weak coefficients. By contrast, the composite index of climate disasters shows a significantly negative coefficient (Model 6), which is consistent with our main results. Other robustness checks also corroborate our findings, as the coefficients on climate risk remain significantly negative.

### 4.3 Moderating Effects of REI Resilience

Despite the significant damage inflicted by climate risk, the resilience of REI may theoretically mitigate these impacts. We analyze the moderating role of REI resilience in climate disaster damage from four perspectives: environmental, institutional, economic, and social.

#### 4.3.1 Environmental Resilience

**FIGURE 4:**
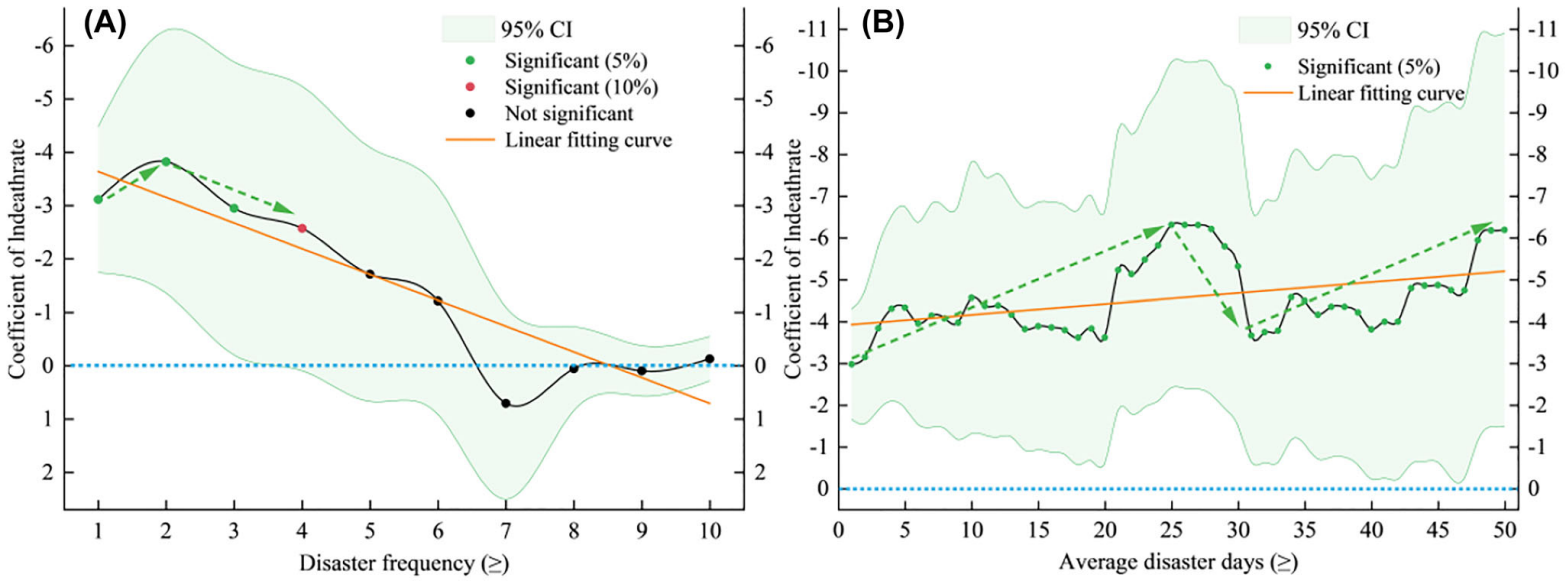
The impacts of climate risk on REI in different external environments (A) disaster frequency and (B) average disaster duration). The sign of the coefficient on the *Y*‐axis has been reversed—the further away and up from the blue 0 line indicates greater climate damage to REI; the same approach is applied to Figure 5.

**FIGURE 5:**
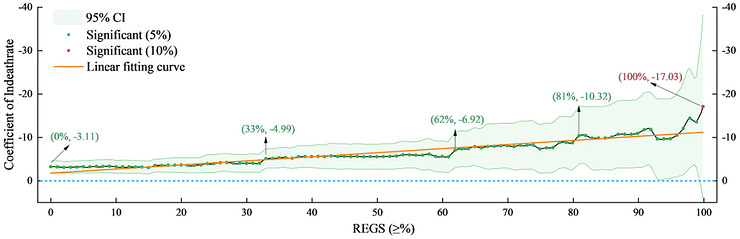
The climate hazards to REI in different internal environments (RE share).

Environmental resilience of REI during disasters can be categorized into external (disaster frequency and average duration) and internal (RE share) factors. We conducted moderation analyses on these indicators (Table 4) and examined the trends of disaster damage alongside variations in these factors (Figures 4 and 5).

**TABLE 4:** The moderating effects of environmental resilience.

	Disaster frequency	Disaster duration	REGS
Model	1	2	3
lnCDR	−2.9878***	−2.4987***	−2.9296***
	(0.663)	(0.593)	(0.623)
Freq	−0.0160		
	(0.040)		
lnCDR × Freq	0.3316^†^		
	(0.207)		
Dura		−0.0015***	
		(0.001)	
lnCDR × Dura		−0.0280***	
		(0.010)	
REGS			0.0197***
			(0.006)
lnCDR × REGS			−0.0613***
			(0.018)
Controls	Yes	Yes	Yes
Country effect	Yes	Yes	Yes
Year effect	Yes	Yes	Yes
Country's time trend	Yes	Yes	Yes
*R*_squared	0.8814	0.8828	0.8830
*N*	1610	1610	1610

*Note*: (1) ***; **; *; † denote significance at 1%, 5%, 10%, and 10.9% levels;“†” indicates 10.9% significance; (2) variables are centralized. (3) REGS is RE generation share.

The moderation coefficient for disaster frequency is 0.33 at the 10.9% significance level, indicating that higher disaster frequency mitigates climate damage to REI. Specifically, REI in countries experiencing more frequent disasters suffers a less negative impact. Figure 4A illustrates that the disaster impact on REI diminishes (brown line) as disaster frequency increases. The damage initially rises with one to two occurrences and then decreases with three to four occurrences, forming an inverted U‐shape. Beyond five occurrences, the damage gradually declines and eventually becomes insignificant. This finding aligns with Carleton and Hsiang (2016), who posited that while increased disaster occurrences may worsen infrastructure damage, countries with high disaster frequency tend to develop stronger resilience, ultimately leading to reduced infrastructure losses.

Model 2 reveals that average disaster duration significantly exacerbates damage, with a moderation coefficient of −0.028^***^. This suggests that prolonged exposure to disasters results in greater harm to REI. The trend chart (Figure 4B) indicates increasing damage to REI (brown line) over time. During the initial period (1–25 days), damage rises gradually with slight fluctuations; in the middle stage (26–31 days), damage decreases, but beyond 31 days, it resumes an upward trend with slight fluctuations, forming an overall upward “N”‐shaped pattern. These findings suggest that while governments may implement disaster mitigation measures, the cumulative effects of disasters (e.g., extreme heat increasing drought risk; heavy rainfall accompanied by storms) may outweigh the benefits of such interventions (Hsiang and Jina 2014).

Model 3 indicates a negative moderation from internal environmental resilience (−0.0613^***^), suggesting that a higher RE share amplifies disaster damages. Figure 5 demonstrates that disaster damage increases with rising RE share. Specifically, damage severity escalates at RE share levels of 33%, 62%, 81%, and 100%, leading to four impact intervals: 0%–32% (very small impact), 33%–61% (relatively small impact), 62%–80% (relatively large impact), and 81%–100% (very large impact). As RE share increases, a country's energy system becomes more reliant on renewable sources, which are inherently intermittent and dependent on natural resources. Consequently, REI in countries with a higher RE share is more vulnerable to climate risk. Notably, the damage for countries with a 100% RE share (−17.03^*^) is nearly six times that of the global average (−3.11^***^ in the baseline model), underscoring the necessity for energy diversity in the face of climate risk.

#### 4.3.2 Institutional Resilience

Table 5 presents moderation effect models and sub‐sample regressions for institutional resilience. The results indicate that RE institutional resilience significantly mitigates the negative impact of climate risk on REI (0.5968^*^), consistent with findings by Agostino (2024) and Chen et al. (2024). The climate disaster damage on REI is insignificant for the low and medium groups. However, in countries with high RE institutional resilience, the impact of climate risk on REI is positive. This suggests that strong institutional resilience not only reduces disaster damage but also enhances REI development, underscoring the critical role of institutional factors.

**TABLE 5:** Moderating effects and quantile regressions of institutional resilience.

Sample	All	Low	Med	High
Model	1	2	3	4
lnCDR	−2.1088^+^	0.8947	0.0535	0.2118^***^
	(1.309)	(1.839)	(0.076)	(0.072)
lnIR	2.7205^*^			
	(1.617)			
lnCDR × lnIR	0.5968^*^			
	(0.351)			
Controls	Yes	Yes	Yes	Yes
Country effect	Yes	Yes	Yes	Yes
Year effect	Yes	Yes	Yes	Yes
Country's time trend	Yes	Yes	Yes	Yes
*R*_squared	0.9483	0.9434	0.9933	0.9929
*N*	952	131	439	355

*Note*: (1) ***; **; *; † denote significance at 1%, 5%, 10%, and 10.7% levels; (2) unlike the traditional tercile method, we employ the mean value ± 0.5 standard deviations as thresholds to account for data distribution characteristics, making the analysis less sensitive to outliers.

#### 4.3.3 Economic Resilience

Table 6 shows that the moderation effect of economic resilience is negative (−1.8051^***^), indicating that economic resilience amplifies the damage of climate risk on REI. Subsample regression results reveal that climate damage is not significant in poor countries. In middle economically developed countries, climate risk significantly damages REI, whereas in developed countries, they promote REI development. Poor countries often prioritize resources for economic and social recovery during disasters and direct investments toward low‐cost energy sources rather than eco‐friendly RE. Similarly, middle‐level countries may pursue rapid urbanization by potentially compromising quality, thus increasing vulnerability to disasters (Rahman 2018). Developed countries, with strong economic resilience, can mobilize sufficient resources to restore REI post‐disaster.

**TABLE 6:** Moderating effects of economic resilience with quantile regressions.

Economic resilience	All	Low	Med	High
Model	1	2	3	4
lnCDR	−1.8105***	−0.0342	−3.4886***	0.3078*
	(0.648)	(0.460)	(1.209)	(0.180)
lnPGDP	0.2682			
	(0.695)			
lnCDR × lnPGDP	−1.8051***			
	(0.470)			
Controls	Yes	Yes	Yes	Yes
Country effect	Yes	Yes	Yes	Yes
Year effect	Yes	Yes	Yes	Yes
Country's time trend	Yes	Yes	Yes	Yes
*R*_squared	0.8830	0.8937	0.8203	0.9988
*N*	1610	716	632	25

#### 4.3.4 Social Resilience

Results in Table 7 indicate that social resilience exacerbates the climate disaster damage, particularly in countries with high social resilience (−2.7796^**^). In contrast, damage in countries with low and medium social resilience is smaller and not significant. Nohrstedt et al. (2022) argue that countries with high social development may face greater disaster risks due to increased exposure.

**TABLE 7:** Moderating effects of social resilience with quantile regressions.

Social resilience	All	Low	Med	High
Model	1	2	3	4
lnCDR	−1.3608**	0.1428	−0.2171	−2.7796**
	(0.560)	(0.391)	(0.343)	(1.136)
HDI	1.2827			
	(5.619)			
lnCDR × HDI	−17.3713***			
	(3.679)			
Controls	Yes	Yes	Yes	Yes
Country effect	Yes	Yes	Yes	Yes
Year effect	Yes	Yes	Yes	Yes
Country's time trend	Yes	Yes	Yes	Yes
*R*_squared	0.8834	0.9315	0.7496	0.9075
*N*	1610	519	496	584

*Note*: ***; **; * denote significance at 1%, 5%, and 10% levels; Abbreviation: HDI, Human Development Index.

### 4.4 Heterogeneity Analysis

A strong REI resilience is generally expected to mitigate climate disaster damage. However, our findings reveal that both economic and social resilience exhibit counterintuitively negative moderation effects. Additionally, the results vary across different economic and social groups, which is primarily due to national heterogeneity in these dimensions (Hsiang and Jina 2014; Song et al. 2024). Consequently, we conducted a deeper analysis of countries at different economic and social levels, examining the moderating roles of environmental and institutional resilience, along with their recovery patterns. The results are presented in Tables 8 and 9.

**TABLE 8:** Environmental and institutional resilience on different economic and social levels.

Moderator	Freq			Dura			REGS			Institution		
PGDP	Low	Med	High	Low	Med	High	Low	Med	High	Low	Med	High
Model	1	2	3	4	5	6	7	8	9	10	11	12
lnCDR	−0.0435	−2.9877***	0.3256*	−0.0136	−2.4436***	0.2655*	−0.0041	−3.2614***	0.2698	0.3318	−0.3718***	0.1719
	(0.497)	(0.973)	(0.167)	(0.496)	(0.897)	(0.148)	(0.440)	(1.069)	(0.209)	(0.314)	(0.134)	(1.101)
*M*	0.0115	−0.0677	0.0062	−0.0001	−0.0023**	−0.0011***	0.0242***	0.0319**	0.0066	−0.0546	0.0485	0.0594
	(0.026)	(0.092)	(0.005)	(0.000)	(0.001)	(0.000)	(0.008)	(0.016)	(0.004)	(0.050)	(0.042)	(0.070)
lnCDR × *M*	−0.0339	0.7224**	0.0914*	0.0020	−0.0419**	0.0034	−0.0304	−0.0630**	−0.0215**	0.3700	1.1118*	−0.0160
	(0.093)	(0.354)	(0.052)	(0.007)	(0.017)	(0.003)	(0.023)	(0.028)	(0.010)	(0.638)	(0.571)	(0.917)
*R*_squared	0.8937	0.8235	0.9988	0.8937	0.8276	0.9988	0.8957	0.8240	0.9988	0.9055	0.9904	0.9916
*N*	716	632	256	716	632	256	716	632	256	418	373	159
Moderator	Freq			Dura			REGS			Institution		
HDI	Low	Med	High	Low	Med	High	Low	Med	High	Low	Med	High
Model	13	14	15	16	17	18	19	20	21	22	23	24
lnCDR	−0.0090	−0.1614	−2.7794***	0.1439	0.6761	−2.0716**	0.2004	−0.0911	−2.7174***	0.4788	0.1843	−0.2591***
	(0.376)	(0.355)	(1.006)	(0.413)	(1.003)	(1.033)	(0.383)	(0.347)	(1.000)	(0.460)	(0.372)	(0.082)
*M*	0.0365	−0.0212	0.0051	−0.0001	0.0018	−0.0023**	0.0133**	0.0436**	0.0182	−0.0600	0.0073	0.0402
	(0.023)	(0.044)	(0.067)	(0.000)	(0.002)	(0.001)	(0.005)	(0.020)	(0.012)	(0.062)	(0.048)	(0.058)
lnCDR ×* M*	−0.3367**	0.0169	0.5472*	0.0005	0.0343	−0.0150	−0.0133	0.0467	−0.0701**	0.6411	−0.1289	0.4498***
	(0.156)	(0.110)	(0.296)	(0.006)	(0.034)	(0.010)	(0.016)	(0.060)	(0.027)	(0.768)	(0.647)	(0.127)
*R*_squared	0.9317	0.7498	0.9086	0.9315	0.7499	0.9089	0.9322	0.7535	0.9100	0.8862	0.9906	0.9923
*N*	519	496	584	519	496	584	519	496	584	294	260	389

*Note*: (1) To simplify the table, control variables, country effect, year effect, and country's time trend are not shown; (2) *M* indicates the moderating variable, for example, Freq, Dura, REGS, and Institutional resilience. (3) ***; **; * denote significance at 1%, 5%, and 10% levels.

Abbreviation: HDI, Human Development Index.

**TABLE 9:** The damage of CDR to renewable energy infrastructure (REI) over time.

Group	Economy			Society		
	Low	Med	High	Low	Med	High
Model	1	2	3	4	5	6
lnCDR	−0.0342	−3.4886^***^	0.3078^*^	0.1428	−0.2171	−2.7796^**^
	(0.460)	(1.209)	(0.180)	(0.391)	(0.343)	(1.136)
Model	7	8	9	10	11	12
L.lnCDR	0.6461^*^	0.2691	−0.1480	0.6272	0.2823	0.4420^*^
	(0.348)	(0.209)	(0.100)	(0.415)	(0.275)	(0.246)
Model	13	14	15	16	17	18
L2.lnCDR	0.7753^*^	0.0966	0.2324^*^	0.5605	0.3511	−1.0434
	(0.436)	(0.319)	(0.122)	(0.409)	(0.608)	(0.788)
Controls	Yes	Yes	Yes	Yes	Yes	Yes
Country effect	Yes	Yes	Yes	Yes	Yes	Yes
Year effect	Yes	Yes	Yes	Yes	Yes	Yes
Country's time trend	Yes	Yes	Yes	Yes	Yes	Yes
Pattern	Build back better	No recovery or recovery to trend	Creative destruction	Uncertain	Uncertain	Recovery to trend

#### 4.4.1 Economic Level

Models 1, 4, 7, and 10 demonstrate that in countries with low economic levels, the climate disaster damage to REI is insignificant, with neither environmental nor institutional resilience altering this outcome.

In middle‐level economic countries, an increased frequency of disasters correlates with reduced damage (Model 2), and RE institutional resilience also mitigates losses (Model 11). However, longer disaster durations and higher RE share lead to greater damage (Models 5 and 8). This indicates that while middle‐level economic countries may learn from disasters and leverage institutional support to restore REI, their response capabilities are limited and vulnerable when disasters are prolonged and when they rely heavily on RE.

For developed countries, climate risk can positively influence REI, with disaster frequency enhancing this effect (Model 3). This suggests that developed countries possess strong adaptive and recovery capacities, as well as the ability to learn from disasters. However, similar to middle‐level economic countries, those with a higher RE share experience greater damage (Model 9), indicating that a heavy reliance on RE can render energy systems more vulnerable to climate risk.

Overall, economic resilience plays a critical role in the capacity of REI to withstand climate disaster exposure, with developed countries exhibiting strong resilience against such risks.

#### 4.4.2 Social Development

The results indicate that disaster frequency exacerbates climate disaster damage in low HDI countries (Model 13). However, other moderating variables, including disaster duration, RE share, and RE policy resilience, do not significantly affect low and middle‐HDI countries (Models 14, 16, 17, 19, 20, 22, 23).

In high HDI countries, disaster frequency alleviates the climate disaster harm (Model 15), reflecting their strong learning capacity in the aftermath of disasters. Additionally, RE institutional resilience similarly mitigates damage (Model 24), suggesting that in socially developed countries, institutional frameworks can effectively enhance the disaster resistance capacity of REI.

In summary, although REI in socially developed countries may still incur climate disaster damage, such damage can be mitigated through increased disaster frequency and robust institutional resilience. Conversely, there is no evidence to support that REI resilience alleviates disaster losses in less socially developed countries.

#### 4.4.3 REI Recovery Patterns

On the basis of the aforementioned framework (Figure 3), we estimated REI recovery patterns over 1 and 2 years by economic and social levels (Table 9). Developed countries show “Creative destruction,” poor ones display “Build back better,” and middle‐level economic countries exhibit either “No recovery” or “Recovery to trend.” For social levels, high social level countries tend to be “Recovery to trend” despite initial damage, whereas low and medium social level countries remain “Uncertain” due to insignificant coefficients.

#### 4.4.4 Geographical Location

As climate risk and RE resources are influenced by natural and geographical conditions, we also conducted heterogeneity analyses based on geographical location and RE types.

Table 10 presents a geographical analysis of the impact of climate risk on REI. South American countries experience the most significant damage (−5.6579^**^). This is followed by Asia (−3.0721^*^) and Africa (−2.2040^**^), whereas Europe shows the least impact (−1.3004^***^). In North America and Oceania, the coefficients are −0.7602 and −1.6043, respectively, but neither is statistically significant.

**TABLE 10:** Heterogeneous effects of climatic disasters on renewable energy infrastructure (REI).

Heterogeneity	Location						RE type			
	Asia	Africa	South America	North America	Europe	Oceania	Water	Solar	Wind	Bioenergy
Model	1	2	3	4	5	6	7	8	9	10
lnCDR	−3.0721*	−2.2040**	−5.6579**	−0.7602	−1.3004***	−1.6043	−2.9881***	−3.1286***	−4.4390***	−3.6256***
	(1.568)	(1.104)	(2.256)	(0.864)	(0.496)	(1.149)	(0.720)	(0.775)	(0.988)	(0.874)
L.lnREI	0.3343**	0.8347***	1.6744***	0.9690***	0.5777***	0.8431***	0.7807***	0.8179***	0.8064***	0.8063***
	(0.165)	(0.111)	(0.639)	(0.223)	(0.141)	(0.134)	(0.105)	(0.031)	(0.036)	(0.048)
Controls	Yes	Yes	Yes	Yes	Yes	Yes	Yes	Yes	Yes	Yes
Country effect	Yes	Yes	Yes	Yes	Yes	Yes	Yes	Yes	Yes	Yes
Year effect	Yes	Yes	Yes	Yes	Yes	Yes	Yes	Yes	Yes	Yes
Country's time trend	Yes	Yes	Yes	Yes	Yes	Yes	Yes	Yes	Yes	Yes
*R*_squared	0.8152	0.9261	0.6751	0.8089	0.9904	0.9748	0.9209	0.8988	0.9137	0.9234
*N*	402	420	162	170	322	135	1610	1610	1610	1610

Note: ***; **; * denote significance at 1%, 5%, and 10% levels. Abbreviation: RE, renewable energy.

**FIGURE 6:**
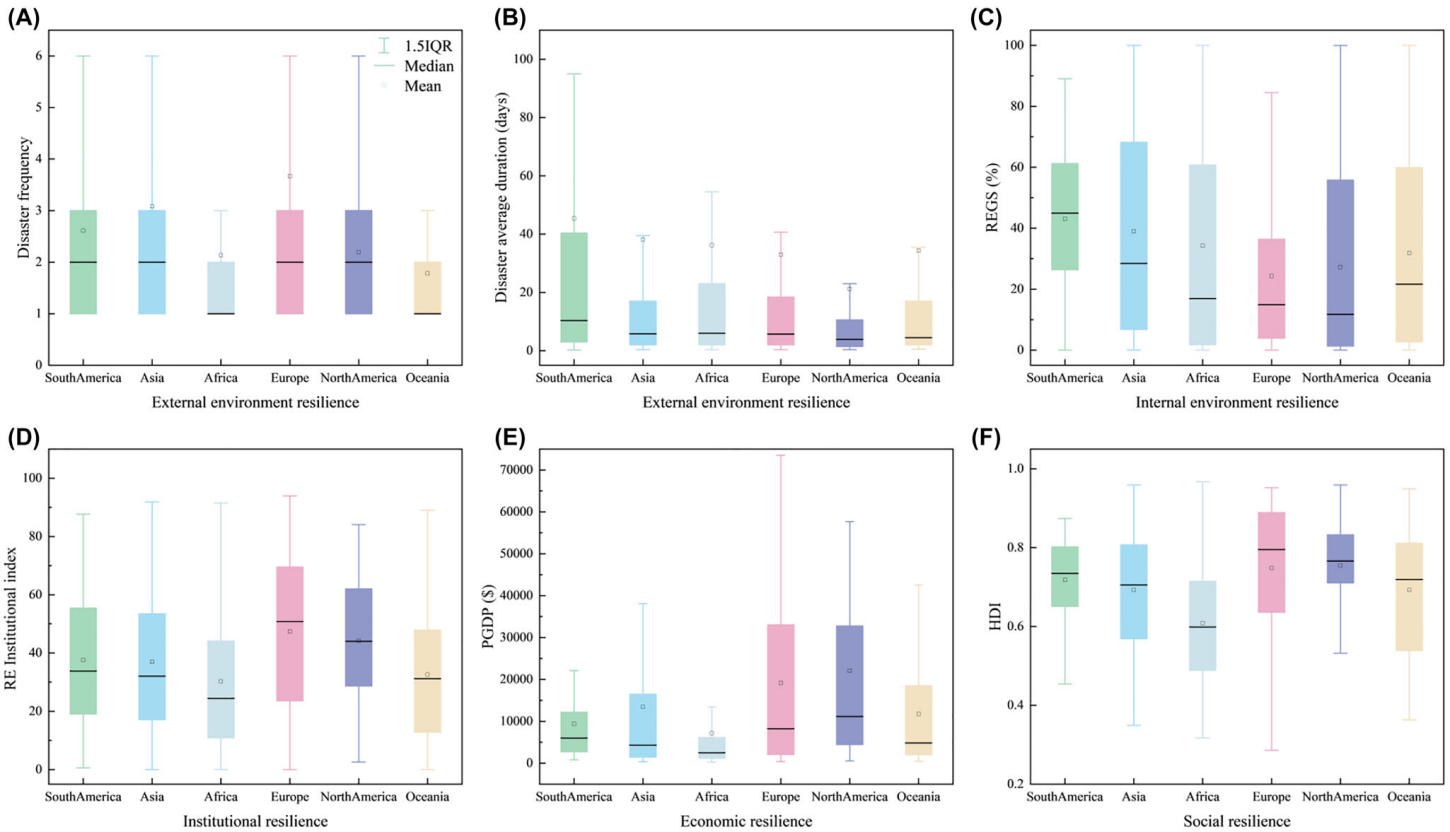
REI resilience of countries. HDI, Human Development Index; RE, renewable energy. Figures A–C show external environment resilience (i.e. disaster frequency and average disaster duration) and internal environment resilience. Figures D–F show institutional, economic and social resilience.

As illustrated in Figure 6E, these disparities may stem from the relatively low economic development levels in South America, Asia, and Africa, which contribute to inadequate infrastructure investment, maintenance, and technology, thereby increasing vulnerability to climate risk. In contrast, North America (particularly the United States and Canada) and Oceania (mainly Australia and New Zealand) benefit from high economic development levels, enabling substantial investment in and maintenance of high‐standard infrastructure.

Moreover, South America significantly surpasses other continents in both disaster duration and RE share (Figure 6B,C), as indicated by the mean and median values. Our earlier findings demonstrate that these environmental factors can exacerbate disaster severity, potentially accounting for South America's pronounced impact. Additionally, the top three most severely affected regions—South America, Asia, and Africa—exhibit significant deficiencies in institutional and economic resilience compared to Europe (Figure 6D,E). However, although Europe excels in many areas, it also experiences the highest average disaster frequency (Figure 6A), which may explain its significant impact from climate risk.

#### 4.4.5 RE Types

All four major RE types are significantly affected by climate risk, with wind energy facing the greatest impact, followed by bioenergy, solar energy, and hydropower. This finding underscores the substantial effects of climate risk across all RE resources, as detailed in Table S1. It highlights the necessity for countries to remain vigilant regarding the impacts of climate risk, irrespective of national heterogeneities.

## 5 Discussions and Implications

Climate disasters, including floods, storms, and extreme temperatures, pose significant threats to existing REI. It not only incurs immediate repair costs but also deters investors and developers from future projects in disaster‐prone areas. Additionally, such disasters disrupt supply chains, transportation, and labor necessary for the construction and maintenance of REI. This study confirms that climate risk significantly hinders REI development through a series of robustness analyses.

Many researchers argue that REI resilience can effectively mitigate disaster damage. By categorizing REI resilience into environmental, institutional, economic, and social aspects, our findings show that the climate disaster damage to REI can indeed be alleviated through institutional resilience and certain environmental factors.

### 5.1 Environment Resilience

The frequency and duration of disasters initially exacerbate the harm caused by climate events to REI; however, as these factors reach a certain threshold, the extent of the damage may decrease. Our results suggest that moderate disaster frequency and duration allow countries to learn from previous disasters and improve predictive awareness, disaster preparedness, infrastructure design, and response strategies (Dai et al. 2015; Neil Adger et al. 2005). Consequently, as external environmental resilience strengthens, damage to REI is alleviated. Furthermore, moderate disasters can stimulate investments in REI. When governments recognize a manageable pattern of recurring disasters, they are more likely to allocate resources toward building resilient infrastructure capable of withstanding future events (Hallegatte 2009).

However, as disaster frequency increases, the moderating effect diminishes, eventually reaching statistical insignificance, whereas the negative impact of disaster duration continues to worsen. This suggests that as disaster frequency escalates, the adaptive capacity of governments and institutions may reach a threshold where the financial, human, and technical resources required to cope with frequent disasters become overstretched. This strain may lead to reduced investments in REI (Chen et al. 2024) or even disruptions in ongoing projects (Agostino 2024). Prolonged disasters can impose long‐term stress on REI, depleting its redundancy and creating complex systemic risks that are challenging to manage. Such risks include continuous depletion of infrastructure resources, supply chain disruptions, population displacement, and cascading failures of infrastructure systems (Agostino 2024). The cumulative effect of these risks can overwhelm even well‐prepared, economically resilient countries (Helbing 2013). Therefore, disaster management plans should prioritize addressing frequent and prolonged disasters, including the development of multi‐layered defense systems.

Our findings indicate that countries with a high dependency on RE are more vulnerable to climate risk, irrespective of their socio‐economic development levels. This highlights the significant disaster risks associated with a heavy reliance on a single category of energy. REI, including wind turbines, solar panels, and hydropower dams, tends to be more exposed to environmental conditions than traditional energy sources (e.g., fossil fuels), making them more susceptible to damage from extreme weather events (Panteli and Mancarella 2015a; Stirling 2010). If a climate disaster disrupts the dominant energy source, the lack of viable alternatives can lead to prolonged power outages (Blazquez et al. 2018). Additionally, the interconnections between energy supply disruptions and broader economic, social, and environmental systems can trigger severe butterfly effects, including unemployment, reduced economic activity, and social unrest, to the extent that even leading European nations might reconsider previously adopted RE measures (Prontera 2021).

Thus, countries, especially those with a high RE share, should not rely solely on RE. Instead, they should invest in a diversified energy mix that includes energy sources less susceptible to climate risk, such as nuclear energy and certain fossil fuels. Furthermore, Esteban and Portugal‐Pereira (2014) confirm the feasibility of 100% RE systems supplemented by battery storage solutions in post‐disaster recovery. Therefore, hybrid RE systems that diversify the energy supply present a viable solution.

### 5.2 Institutional Resilience

Our findings indicate that institutional resilience not only mitigates climate disaster damage to REI but also fosters its development in countries with high RE institutional resilience. This suggests that institutional resilience plays a critical role in withstanding, recovering from, and advancing REI post‐disaster, consistent with the conclusions of Khan et al. (2022). A stable political environment ensures effective implementation of RE support policies (Chen et al. 2024), thereby reducing the adverse impacts of climate change on RE systems (Shahbaz et al. 2022).

Specifically, high institutional resilience typically encompasses robust post‐disaster recovery frameworks, including financial aid, reconstruction incentives, and technical support for damaged REI. These frameworks not only facilitate the restoration of damaged infrastructure but also create opportunities for upgrades and resilience enhancements, resulting in advanced and disaster‐resistant REI (Hallegatte et al. 2016). Furthermore, stable, effective, and flexible policy frameworks during and after disasters can foster institutional innovation (Chen et al. 2024) and encourage continuous investment (Porter et al. 2015).

### 5.3 Economical Resilience

Our analysis reveals that the initial damage from climate risk is insignificant for poor countries but significant for middle‐level economic countries. Much of the existing literature suggests that poorer regions are more vulnerable to environmental shocks (Hallegatte and Rozenberg 2017), whereas wealthier regions tend to be better protected (Hallegatte et al. 2013). Hallegatte et al. (2020) note that poor populations, although highly vulnerable, often record small asset losses simply because there is less to lose. Uzar (2020) argues that economic capacity constraints hinder the uptake and implementation of RE support schemes, limiting the effectiveness of formal incentives. These studies imply that limited economic capacity reduces both the exposure of poor populations and the effectiveness of formal policy incentives, thus helping explain why, in our sample, environmental and institutional resilience did not enhance REI in poor countries. However, Felbermayr and Gröschl (2014) found that only developed countries experience negative impacts from droughts and storms. Rahman (2018) argues that poor countries often construct temporary and vulnerable infrastructure to meet minimum needs, while rapidly developing middle‐level economic countries may compromise infrastructure quality due to cost constraints. Our findings regarding REI support this argument, indicating the need for governments to prioritize the quality and resilience of REI rather than merely increasing its quantity. In middle‐level countries, high disaster frequency is associated with low REI damage, and strong institutional resilience further mitigates losses, whereas long disaster duration and high RE share increase damage. The frequency–learning channel aligns with global evidence that repeated shocks sometimes catalyze policy and capability upgrading once state capacity crosses a threshold (Skidmore and Toya 2002). The institutional result is consistent with evidence that credible, well‐designed RE policies (e.g., feed‐in tariffs) accelerate RE deployment and associated capability building (Jenner et al. 2013), yet other work highlights the heterogeneous importance of institutional resilience in shaping the effect of disaster experience on adaptation (Nohrstedt et al. 2022). By contrast, the damage from long disaster durations and high RE share aligns with power‐system evidence that prolonged events and high‐penetration variable renewables increase compounding failures and meteorological sensitivity (Panteli and Mancarella 2015b) unless supported by adequate flexibility (Van Der Wiel et al. 2019). Therefore, resilience strategies in low‐income countries should focus on building capacity and expanding access to finance to enable institutional tools, such as tariffs, auctions, and standards, to take hold.

Despite insignificant initial damage, climate risk in poor countries promoted REI growth over the following 2 years, reflecting a “Build back better” pattern. This likely results from underdeveloped REI, making it easier to install modern systems that surpass pre‐disaster levels. In contrast, medium‐level economic countries are more likely to focus on restoring existing infrastructure, leading to “Recovery to trend” or “No recovery” patterns.

Notably, climate risk can promote REI development in developed countries, underscoring the importance of economic resilience in enhancing the risk resistance of REI. These countries take advantage of the financial and technical resources (Noy 2009) necessary to construct high‐quality, disaster‐resistant REI. The influx of international aid and increased attention post‐disaster often provide opportunities for infrastructure upgrades rather than simple restoration (Carley et al. 2017). This finding aligns with Hsiang and Jina (2014)’s “Creative destruction” hypothesis, highlighting the role of economic development in post‐disaster recovery. However, even in economically resilient countries, a high RE share can create vulnerabilities, as renewable sources, particularly solar and wind energy, are sensitive to extreme weather events. In the absence of a diversified energy mix or adequate storage systems, climate risk can lead to significant disruptions. Therefore, these countries should aim to diversify their energy mix, integrating RE with other sources to ensure backup during extreme weather events.

### 5.4 Social Resilience

Our analysis reveals that social resilience can exacerbate the climate disaster damage to REI. This finding aligns with the empirical results of Khribich et al. (2021) and Nohrstedt et al. (2022), who argue that social development does not significantly contribute to RE development. High social resilience indicates a deeply integrated and complex socio‐economic system. This interconnectedness means that climate risk not only damages REI but also triggers severe cascading effects on society and the economy, thereby exacerbating the damage. However, high‐social‐level countries recover significantly within 1 year, showing a “Recovery to trend.” We also observe that in these countries, disaster frequency and institutional support can significantly mitigate this damage. This is consistent with findings from Nohrstedt et al. (2022), which suggest that wealthier nations, despite facing greater disaster exposure risks, possess better governance capabilities for disaster recovery. This coheres with Aldrich (2011)’s research showing that denser social capital and stronger civic networks predict more effective post‐disaster recovery. Consequently, the disaster experience accumulated from frequent exposure and institutional advantages can effectively enhance responses to climate events in socially cohesive contexts. In contrast, weak social cohesion in low‐ and medium‐social‐level countries may hinder recovery, leading to uncertain outcomes.

## 6 Conclusions

Accelerating global climate risk poses increasing threats to REI. The heterogeneous impacts of climate risk on REI across different countries and the moderating role of REI resilience have not been empirically analyzed. To address these issues, this study established dynamic panel empirical models based on a dataset encompassing 215 countries and regions from 2004 to 2022, yielding the following main conclusions:

1. Climate risk significantly impedes global REI development, as confirmed by various robustness analyses.
2. The climate disaster damage to REI is mitigated by the disaster frequency. As frequency rises, climate damage increases, then decreases, ultimately becoming insignificant, revealing an inverted U‐shaped relationship. Differently, the average duration of disasters exacerbates climate disaster damage, initially increasing and then fluctuating upward, resembling an “N” trend. Additionally, as the RE share increases, the damage intensifies, progressing through four increasingly severe stages.
3. Institutional resilience mitigates climate disaster damage to REI. Notably, in countries with high RE institutional resilience, climate risk can promote REI development.
4. Economic resilience, while often amplifying global climate disaster impacts, can foster REI growth in developed countries, resulting in a “Creative destruction” recovery pattern, especially when disaster frequency and duration intensify this effect. For poor countries, disasters can similarly promote long‐term REI growth despite insignificant initial damage, showing a “Build back better” pattern.
5. Social resilience tends to exacerbate climate disaster damage, particularly in high‐social level countries, but disaster frequency and institutional resilience can mitigate this effect, fostering a “Recovery to trend” pattern within 1 year.
6. South American countries experience the most significant climate disaster damage to REI, followed by Asia and Africa, with Europe being the least affected. Additionally, wind energy infrastructure is the most impacted, followed by bioenergy, solar energy, and hydropower.

This article analyzes the moderating roles of REI resilience and the heterogeneity across different economic and social conditions. However, the interactions among the driving factors of REI development may be more complex than captured in this analysis. Future research should consider methods capable of handling high‐dimensional data and analyzing causal complexity, such as machine learning or qualitative comparative analysis.

## Author Contributions

**Jingke Hong**: investigation, data curation, writing – original draft preparation. **Yang Chen**: methodology, software, writing – reviewing and editing. **Wen Yi**: conceptualization, supervision, validation, writing – reviewing and editing. **Hung‐lin Chi**: writing – reviewing and editing.

## Conflicts of Interest

The authors declare no conflicts of interest.

## Supporting information




**Supporting Information Table S1**: How climate disasters affect REI. **Supporting Information Table S2**: Interactions between climate disasters.

**Supporting Information**: risa70273‐supp‐0002‐SuppMat.rar

## Data Availability

The data that support the findings of this study are available from the corresponding author upon reasonable request.

## Endnotes

1 We also estimated models with a 2‐year lag, which yielded consistent results, but for brevity, these are not reported here.

2
https://www.emdat.be

3
https://tinyurl.com/2ay2xdjv
