# Supplementary material for: Global Renewable Energy Infrastructure Resilience Under Climate Risks
Source: Risk Anal. 2026 Jun 23;46(7):e70273. doi: 10.1111/risa.70273 (PMC13289525; doi:10.1111/risa.70273)
Supplement: Supplementary file 1 — Supporting Information Table S1: How climate disasters affect REI. Supporting Information Table S2: Interactions between climate disasters. [file RISA-46-0-s001.docx]

**Supplementary Information**

**Global renewable energy infrastructure resilience under climate risks**

Various components of REI systems are susceptible to climate disasters, categorized into direct physical damage and indirect cascading damage (Table S1). Direct damages include harm to core components, auxiliary systems, transmission infrastructure, and raw materials and fuel supplies (Agostino, 2024). Indirect impacts involve a) Mutual Exacerbation of Natural Disasters: For example, extreme high temperatures can increase water evaporation and reduce soil moisture, heightening drought risk; storms often bring heavy rainfall, leading to flooding (Table S2). b) Socio-Economic and Institutional Impacts: These affect the recovery capacity of facilities.

Table S1. How climate disasters affect REI

| Climate disasters/REI | Solar Energy | Wind Energy | Hydropower | Bioenergy |
| --- | --- | --- | --- | --- |
| Flood | Inundation of photovoltaic panels, support structures, and inverters, potentially leading to short circuits or permanent damage.  Submersion or severance of cable networks, affecting grid connectivity.  Flooding of access roads and operational areas, impeding maintenance access and equipment transportation, reducing resilience.  Secondary hazards like landslides and mudflows can directly compromise solar power station infrastructure. | Erosion of wind turbine foundations can lead to destabilization or collapse.  Submersion and damage to control equipment, transformers, and transmission lines.  Flooding obstructs routine maintenance. | Exceeding the design parameters of dams can result in dam failures or reservoir leakages.  Sedimentation in water channels reduces generation efficiency.  The inundation of powerhouses and electromechanical equipment causes irreversible damage. | Destruction of agricultural land, leading to compromised biomass sources and raw material loss.  Inundation of storage facilities, degrading feedstock.  Contamination of anaerobic digesters, disrupting fermentation and damaging equipment. |
| Storms | Structural damage or displacement of solar panel arrays due to high winds.  Malfunction of tracking systems in extreme wind conditions.  Mechanical damage from wind-borne debris or vegetation.  Disruption of grid connectivity due to damaged cable networks.  Dust storms and hail obstruct solar irradiance. | Extreme winds can deform or fracture turbine blades, and damage nacelles and electrical components.  Vulnerability of off-site transmission lines and substations to storm damage. | Storm-induced waves stress dam structures and spillways.  On-site transmission lines and substations are vulnerable to storm damage. | Destruction of trees, crops, and other biomass resources, reducing bioenergy fuel supply.  Degradation of biomass quality, including moisture increase and decomposition.  Damage to bioenergy plant structures, equipment, and transmission lines, disrupting generation. |
| Drought | Dust accumulation on photovoltaic surfaces, reducing generation efficiency.  Water scarcity hinders cleaning operations for photovoltaic modules.  Overheating of modules due to decreased atmospheric humidity. | Alteration in regional pressure distribution, affecting wind speed and turbine generation efficiency.  Reduced air density impacts energy capture by turbines.  Frequent maintenance shutdowns to prevent overheating during drought-associated high temperatures. | Reduced water levels in reservoirs curtail hydroelectric generation.  River depletion during droughts can eliminate generation capacity altogether. | Reduction in biomass feedstock growth.  Decline in wetlands and pastures productivity, affecting biogas feedstock supply. |
| Extreme temperatures | High temperatures reduce photovoltaic cell efficiency and accelerate degradation.  Low temperatures can cause ice formation on panels, blocking solar irradiance.  Extreme cold slows electrochemical reactions, reducing power output. | High temperatures reduce efficiency and increase turbine failure risks.  Low temperatures cause ice formation on blades, affecting performance and increasing structural loads. | Accelerated water evaporation lowers water levels in reservoirs, reducing hydropower generation capacity.  Thermal expansion and contraction of dam materials due to temperature extremes could compromise dam structural integrity.  Droughts or storms linked to extreme temperatures can indirectly affect water availability for hydropower.  Ice formation in water intakes and diversion structures can obstruct water flow and hinder generation. | Suppression of biomass growth under extreme temperatures.  Accelerated decomposition of biomass in high temperatures.  Ice formation impedes harvesting and transport operations during low temperatures. |

Notes: Floods are mainly caused by heavy rain, glacier melting, hurricanes, etc.; Extreme temperatures include extremely high temperatures and extremely low temperatures.

Appendix Table S2. Interactions between climate disasters

| Impact on/Affected by | Drought | Extreme Heat | Extreme Cold | Flood | Heavy Rainfall | Storms |
| --- | --- | --- | --- | --- | --- | --- |
| Drought | —— | Increases extreme heat risk:  Reduced soil moisture leads to vegetation decrease, diminishing evapotranspiration and subsequently elevating surface temperatures | Rare correlation | Increases flood risk:  Vegetation reduction due to drought causes soil crusting, reducing infiltration capacity. Consequently, when heavy rainfall occurs, runoff increases, potentially leading to flooding | Rare correlation | Rare correlation |
| Extreme Heat | Increases drought risk: Extreme temperatures increase water evaporation. | —— | Rare correlation | Rare correlation | Increases rainfall risk:  Extreme heat increases atmospheric water vapor content and enhances air convection | Increases storm risk:  Temperature gradient changes can potentially trigger strong winds |
| Extreme Cold | Rare correlation | Rare correlation | —— | Increases flood risk:  Formation of ice and snow, prone to melting during temperature rise | Increases rainfall risk:  Cold air masses encountering warm, moist air flows can potentially produce precipitation | Increases storm risk:  Temperature gradient changes can potentially trigger strong winds |
| Flooding | May alleviate | May alleviate | Rare correlation | —— | May coincide:  Heavy rainfall inducing flooding | May coincide:  Potential to induce localized air current variations |
| Heavy Rainfall | May alleviate | May alleviate | Rare correlation | Increases flood risk:  Intense precipitation | —— | May coincide:  Associated with strong convective weather systems |
| Storms | Increases drought risk:  Vegetation damage and accelerated water evaporation | May alleviate | Increases cold risk:  Potential to bring cold waves | Increases flood risk:  Storm surges and intense precipitation | May coincide:  Influences moisture transport and precipitation distribution | —— |

**Reference**

Agostino, M., 2024. Extreme weather events and firms’ energy practices. The role of country governance. Energy Policy 192, 114235. https://doi.org/10.1016/j.enpol.2024.114235
